# MiSelect R System: the validation of a new detection system of CTCs and their correlation with prognosis in non-metastatic CRC patients

**DOI:** 10.1038/s41598-023-31346-9

**Published:** 2023-03-23

**Authors:** Chun-Chi Lin, Chih-Yung Yang, Tzu-Chao Hung, Chun-Hung Wang, Sheng-Wen Wei, Perry Schiro, Ju-Yu Tseng, Chi-Hung Lin, Jeng-Kai Jiang

**Affiliations:** 1 Division of Colon and Rectal Surgery, Department of Surgery, Taipei Veterans General Hospital, No.201, Shipai Rd. Sec. 2, Beitou Dist., Taipei City, 112 Taiwan; 2 Department of Teaching and Research, Taipei City Hospital, Taipei City, 104 Taiwan; 3 MiCareo Taiwan Co., Ltd., 5F., No. 69, Ln. 77, Xing Ai Rd., Neihu Dist., Taipei City, 114 Taiwan; 4 Department of Biological Science and Technology, National Yang-Ming Chiao-Tung University, Hsinchu, 300 Taiwan; 5 Institute of Microbiology and Immunology, National Yang-Ming Chiao-Tung University, Taipei City, 112 Taiwan; 6 Cancer Progression Research Center, National Yang Ming Chiao-Tung University, Taipei City, 112 Taiwan; 7 School of Medicine, National Yang-Ming Chiao-Tung University, Taipei City, 112 Taiwan

**Keywords:** Biological techniques, Cancer

## Abstract

Circulating tumor cells (CTCs) in blood are accepted as a prognostic marker for patients with metastatic colorectal cancer (CRC). However, there is limited data on the use of CTCs as a prognostic marker for non-metastatic patients. In the current study, we used a rare cell automated analysis platform, the MiSelect R System, to enumerate CTCs from blood in non-metastatic CRC patients, and corelated the number of CTCs with the clinical staging and survival. The presence of CTCs in mesenteric vein blood (MVB) samples from 101 CRC patients was significantly associated with T stage. Patients with 1 or more CTCs per 8 mL of MVB exhibited significantly worse disease-free survival (DFS) and cancer-specific survival (CSS) compared to patient without CTCs. The presence of CTCs before surgery is an independent marker for both DFS and CSS. CTC presence after surgical resection is also a prognostic marker. CTCs are a potentially useful prognostic and predictive biomarker in non-metastatic CRC patients that may further stratify patient’s risk status within different stages of disease.

## Introduction

Colorectal cancer (CRC) is one of the leading causes of death worldwide^1^. The high mortality of colorectal cancer patients is tightly linked to low adherence to screening campaigns, late diagnosis, and distant metastases^2,3^. Approximately half of patients will develop distant metastasis after CRC resection, usually leading to poor prognosis^4^. In clinical practices, adjuvant chemotherapy is used in stage III and high-risk stage II CRC patients to eradicate the occult micrometastatic tumor cells before metastatic disease becomes clinically evident^5^. Patients classified with TNM above stage IIB, elevated preoperative carcinoembryonic antigen (CEA), angio-invasive growth, tumor perforation or obstruction and insufficient lymph node sampling (< 10 nodes) are eligible for adjuvant therapy^6^. Some patients receive adjuvant therapy without having presumed micro-metastasis because of the lack of high sensitivity and specificity of the known risk factors for disease recurrence. Other patients are classified as having a low risk for disease recurrence and do not receive adjuvant therapy, but still progress with recurrence. The early detection of micrometastases could identify patients who are most (and least) likely to benefit from adjuvant therapy. There is an unmet clinical need in disease management for better tools, reliable biomarkers, and simpler tests for early detection of disease recurrence or micro-metastasis.

Metastasis is a multistep process caused by dissemination of malignant cells from the primary tumor^7^. Cancer cells which undergo intravasation result in circulating tumor cells (CTCs) in the bloodstream or lymphatic system and carry the potential for metastatic tumor formation at distant sites^8^. Several studies have shown that the number of CTCs correlates with progression-free survival and overall survival in metastatic CRC patients^9–12^. These studies were primarily conducted with stage IV patients where distant metastasis had already occurred, providing limited evidence in understanding if CTC count predisposes the development of metastasis. The variability of non-standardized detection methods for CTCs have made inter-study comparisons difficult resulting in limited data about the prognostic role of CTCs in non-metastatic CRC patients^13–17^. Because of the extremely low frequency of CTCs in non-metastatic CRC, some studies have used larger volumes of peripheral blood to analyzed the correlation between CTC number and outcome ^16,18^. Mesenteric vein blood obtained during surgery showed higher CTC amounts than found in peripheral blood^19^. In this study, mesenteric vein blood and peripheral blood were both used in a CTC enumeration assay with a demonstrated enhancement of the sensitivity of CTC detection.

CTC isolation remains a challenge due to the scarcity and heterogeneity of these cells in blood. Label-free approaches to cell isolation, such as by size, typically suffer from low recovery, clogging of filters, complicated integration of external force fields, low cell purity, and loss of smaller rare cells limiting their broad utility^20^. Antibody-based methods to isolate rare cells based on expression of surface marker proteins typically use immunomagnetic isolation by magnetic fields using antibodies immobilized to magnetic beads^21^; microfluidics approaches with antibodies immobilized on a microfluidic chip^22^; or fluorescence-activated methods where rare cells are detected and sorted based on laser-induced fluorescence of fluorophore-labeled antibodies^23^. A new developed microfluidic platform, for CTC isolation, antibody labeling, and fluorescence imaging, allows for consistent measurement over the whole study period. This study validated the accuracy, linearity, limits of blank and detection, and the reproducibility of this microfluidic platform. It also explored the prognostic value of CTC presence before and after surgical resection in patients with potentially curable disease, focusing on non-metastatic CRC.

## Results

### Analytical validation of CTC enumeration assay

The assay accuracy is defined as the recovery percentage of a known number of added cultured SkBr3 cells across a range that covers clinically meaningful concentrations of CTCs. Instead of estimating cell counts using serial dilutions, which give wide variability, we pipetted individual cells to add an exact number for a better simulation of the rare number of CTCs found in early-stage disease. Either 1024 cells, 512 cells, 128 cells, 32 cells, 8 cells, or 1 cell were added into 8 mL healthy donor blood for each experiment. The overall recovery rate efficiency was defined as the recovered cell numbers observed as a proportion of the spiked cell numbers. The average overall recovery rate was 91.3% ± 10.3% across the entire spiked cell range (Supplemental Fig. 1A and Supplemental Table 1). In 50 experiments where only 1 cell was spiked into 8 mL of blood, that single cell was recovered 98% of the time. The assay linearity was measured by plotting observed cell counts as a function of spiked in cell counts across various concentrations. The assay was shown to be linear across all spiked concentrations from 1 to 1024 cells with a slope of 0.902 and R^2^ of 0.994 (Supplemental Fig. 1B). To explore the detection efficiency for CTCs that express low levels of EpCAM, e.g., by undergoing epithelial-to-mesenchymal transition (EMT), we used cultured MDA-MB-231 as an EpCAM^low^ cell model to spike into blood in three concentration groups: 256 cells, 40 cells and 16 cells. The average overall recovery rate was 80% ± 8.6% across the entire spiked cell range (Supplemental Fig. 2).

The limit of background (LOB) is defined as the highest CTC count expected to be found when replicates of a blank sample containing no CTCs were tested. We tested replicates of twenty-eight healthy subject’s blood samples and found no CTCs, with a resulting LOB of zero CTCs (Supplemental Table 2). The limit of detection (LOD) is defined as the CTC count for which the probability of falsely claiming the absence of a CTC is 5%, given a 5% (or lower) probability of falsely claiming the presence of a CTC. We tested fifty replicates with only 1 cell spiked into blood and in 49 out of 50 tests we recovered 1 cell. From these 50 tests, the probability of falsely claiming the absence of a CTC was 2%, resulting in the LOD of the assay being 1 CTC in 8 mL of blood (Supplemental Table 1).

The precision of the MiSelect R System was assessed by evaluating variation in overall recovery efficiency for triplicate samples of SkBr3 cells spiked in blood. To better characterize assay precision, recovery efficiency was further evaluated across twenty days and among three different operators. Triplicate contrived samples of a known number of spiked SkBr3 cells in blood for two concentrations (1024 cells defined as high and 32 cells defined as low) were processed. The recovered cells were counted on the MiSelect R System to determine the overall recovery efficiency (Supplemental Table 1). The coefficient of variation (CV) for the triplicate samples was 7.82% for the high cell concentration and 9.23% for the low cell concentration.

### Identification of CTCs in patients with non-metastatic CRC and tubular adenoma in peripheral blood and mesenteric vein blood

**Figure 1:**
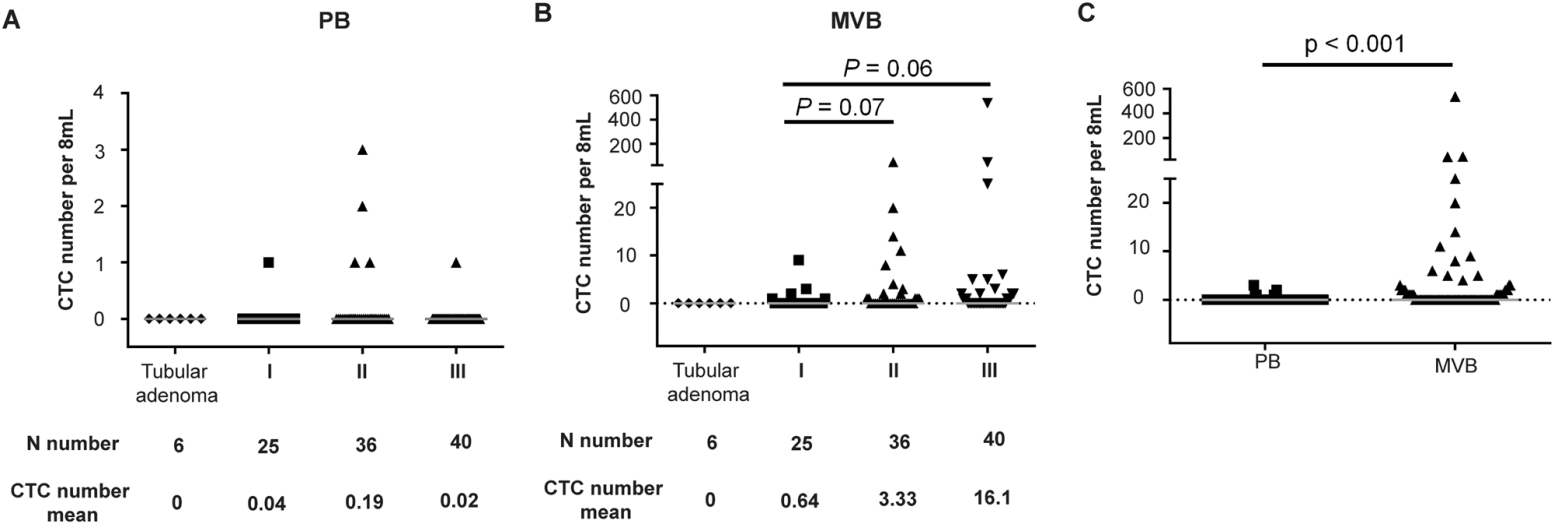
The number of CTCs found in patients with tubular adenoma and at different stages of CRC in PB (**A**) or MVB (**B**). The Y axis uses a log scale to show the range of CTC counts. The p-value shows the statistical difference between the stage I patients and all of the other stages. CTC amount is higher in MVB than PB samples (**C**).

The main clinicopathological characteristics of the 101 CRC patients were presented in Table 1. CTC number and detection rate of patients with non-metastatic CRC and tubular adenoma in peripheral blood (PB) or mesenteric vein blood (MVB) were shown in Fig. 1A and B. CTCs were not found in 6 patients with tubular adenoma in PB or MVB. In contrast, CTCs were found preoperatively in 6 of 101 patients (5.9%) in PB samples and in 37 of 101 patients (36.6%) in MVB samples. The number of CTCs in the CRC patients was higher than that in the tubular adenoma group (0 verse 0.09 cells per 8 mL PB and 0 verse 7.7 cells per 8 mL, respectively.) The average number of CTCs was positively correlated with staging in MVB (Fig. 1B), with the number of CTCs being significantly higher in MVB samples than PB samples (Fig. 1C).

**Table 1:** Clinical demographic data.

Characteristics	Total (N = 101)	CTC present in PB or MVB (N = 39)	CTC absent in PB or MVB (N = 62)	*P* value
Median age, years (range)	65 (37–87)	65 (37–75)	64 (37–87)	
Sex, n (%)				
Male	58 (57.4%)	23 (59.0%)	35 (56.5%)	0.84
Female	43 (42.6%)	16 (41.0%)	27 (43.5%)	
Location of primary tumor, n (%)				**0.048**
Right-side Colon	29 (28.7%)	12 (30.8%)	17 (27.4%)	
Left-side Colon	45 (44.6%)	12 (30.8%)	33 (53.2%)	
Rectum	27 (26.7%)	15 (38.5%)	12 (19.4%)	
T stage, n (%)				**0.009**
T1	18 (17.8%)	3 (7.7%)	15 (24.2%)	
T2	15 (14.9%)	2 (5.1%)	13 (21.0%)	
T3	42 (41.6%)	22 (56.4%)	20 (32.2%)	
T4	26 (25.7%)	12 (30.8%)	14 (22.6%)	
N stage, n (%)				0.796
N0	61 (60.4%)	22 (56.4%)	39 (62.9%)	
N1	30 (29.7%)	13 (33.3%)	17 (27.4%)	
N2	10 (9.9%)	4 (10.3%)	6 (9.7%)	
Stage, n (%)				0.081
I	25 (24.8%)	5 (12.8%)	20 (32.3%)	
II	36 (35.6%)	17 (43.6%)	19 (30.6%)	
III	40 (39.6%)	17 (43.6%)	23 (37.1%)	
Vascular invasion, n (%)				0.61
Present	20 (19.8%)	9 (23.1%)	11 (17.7%)	
Absent	75 (74.3%)	29 (74.3%)	46 (74.2%)	
No data	6 (5.9%)	1 (2.6%)	5 (8.0%)	
Lymphatic invasion, n (%)				1.0
Present	25 (24.8%)	10 (25.6%)	15 (24.2%)	
Absent	70 (69.3%)	28 (71.8%)	42 (67.8%)	
No data	6 (5.9%)	1 (2.6%)	5 (8.0%)	
Perineural invasion, n (%)				0.532
Present	4 (4.0%)	1 (12.8%)	3 (7.5%)	
Absent	91 (90.1%)	37 (84.6%)	54 (83.5%)	
No data	6 (5.9%)	1 (2.6%)	5 (8.0%)	
Preoperative CEA, n (%)				0.181
< 5 ng/mL	66 (65.3%)	22 (56.4%)	44 (71.0%)	
≥ 5 ng/mL	34 (33.7%)	16 (41.0%)	18 (29%)	
No data	1 (1.0%)	1 (2.6%)	0 (0%)	
Preoperative CA-199, n (%)				0.129
< 37 IU/mL	87 (86.1%)	31 (79.5%)	56 (90.3%)	
≥ 37 IU/mL	12 (11.9%)	7 (17.9%)	5 (8.1%)	
No data	2 (2.0%)	1 (2.6%)	1 (1.6%)	
MSI status (%)				0.126
MSI-H	8 (7.9%)	5 (12.8%)	3 (4.8%)	
MSS	80 (79.2%)	28 (71.8%)	52 (83.9%)	
No data	13 (12.9%)	6 (15.4%)	7 (11.3%)	
Number of nodes sampled				0.171
< 12	7 (6.9%)	1 (2.6%)	6 (9.7%)	
≥ 12	94 (93.1%)	38 (97.4%)	56 (90.3%)

### Association of CTCs with clinicopathological parameters in CRC

To investigate whether the detection of CTCs was associated with clinicopathological parameters, we first analyzed the correlation to the presence of any CTCs (≥ 1). Table 1 shows the patients’ characteristics divided between patients without any CTCs and those with 1 or more CTCs. CTC positivity was correlated with T stage and the location of primary tumor. There was no significant difference in the other patient characteristics according to CTC positivity.

### Survival outcomes analysis according to CTC positivity in PB or MVB prior to surgery

**Figure 2:**
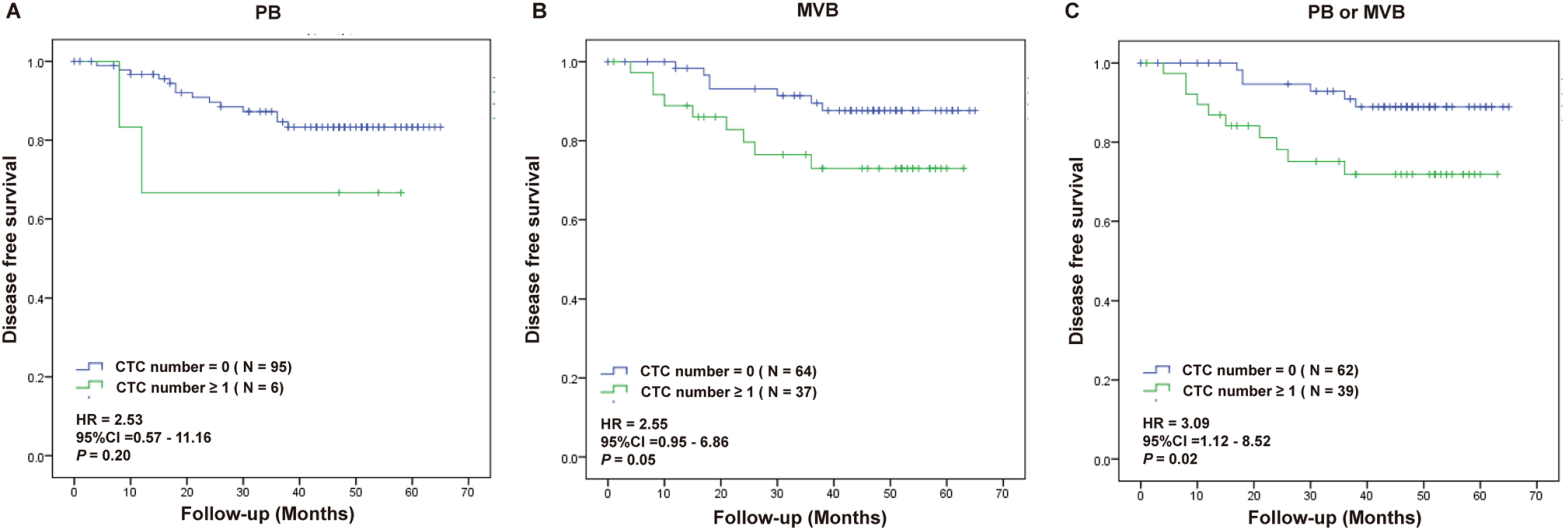
Kaplan-Meier survival analysis for DFS in patients with one or more CTC in PB (**A**), MVB (**B**) or either one (**C**) samples before surgical operation.

**Figure 3:**
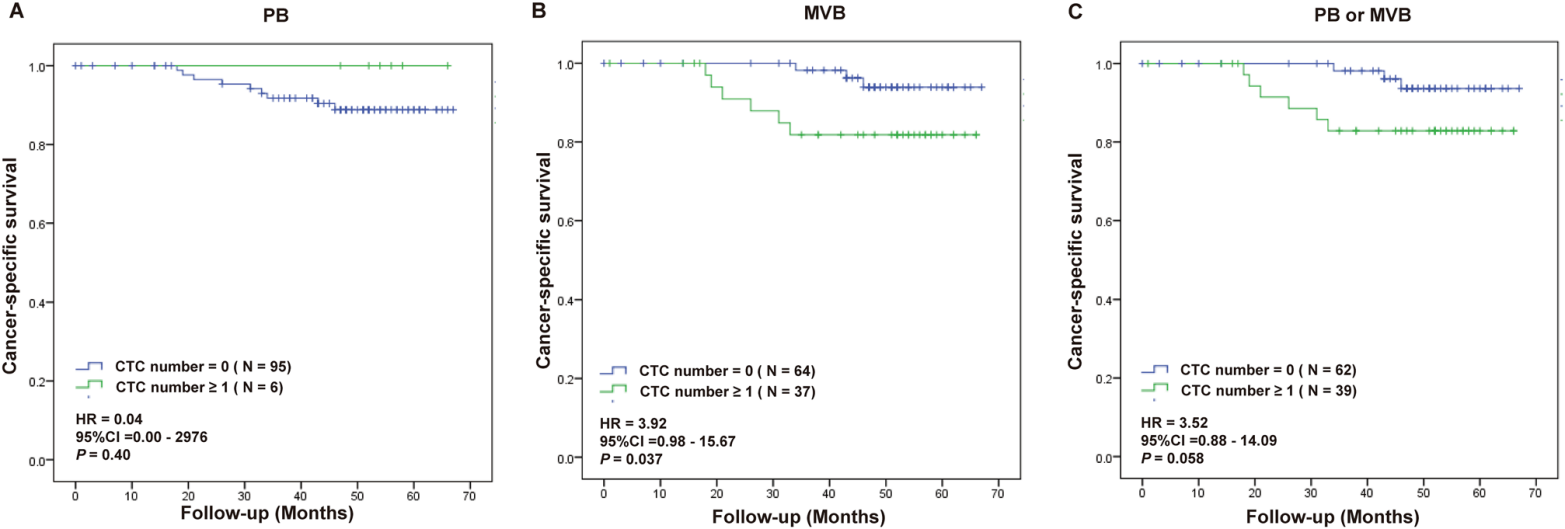
Kaplan-Meier survival analysis for CSS in patients with one or more CTC in PB (**A**), MVB (**B**) or either one (**C**) samples before surgical operation.

All patients were categorized into CTC-positive (CTC number ≥ 1) and CTC negative (CTC number = 0) groups based on their sample status prior to surgery. To investigate the relationship between CTCs and clinical outcomes, we analyzed the survival outcomes (disease free survival (DFS) and cancer-specific survival (CSS)) relative to CTC positivity in PB or MVB in non-metastatic CRC patients before surgery. The average follow-up period was 63.2 months with 16 (15.8%) cases of recurrence and 9 (8.9%) cases of cancer related death. Worse DFS was found when one or more CTC was detected in MVB (Fig. 2B). Significantly worse DFS was also found when at least one CTC was found in either PB or MVB (Fig. 2C). For the six patients where CTC were only found in PB, the DFS appeared worse but it not significantly different with a *P*—value of 0.2 (Fig. 2A). At least one CTC detected in MVB demonstrated significant difference for worse CSS (Fig. 3B). There was no significant difference for CTC positivity in PB (Fig. 3A). Worse CSS was also found when at least one CTC was found in either PB or MVB (Fig. 3C). Table 2 showed the multivariate Cox analysis on various factors for DFS and CSS in CRC patients. Other than CTC status, only the serum level of CA-199 showed significance for CSS.

**Table 2:** Multivariate analysis for DFS and CSS.

	DFS				CSS		
	HR	95% CI	*P* value		HR	95% CI	*P* value
CTC presence in MVB or PB	3.00	1.046–8.616	**0.041**		6.47	1.074–38.95	**0.042**
Age	1.017	0.351–2.946	0.975		5.69	0.713–45.50	0.101
TNM Stage	1.459	0.642–3.313	0.367		1.482	0.397–5.53	0.558
Serum CEA	0.926	0.303–2.829	0.893		0.256	0.036–1.802	0.171
Serum CA199	2.302	0.67–7.91	0.186		12.41	1.642–93.88	**0.015**
Tumor Location	0.71	0.368–1.371	0.308		0.983	0.347–2.781	0.974
MSI Status	0.322	0.035–2.919	0.314		0	0	0.986
Number of nodes sampled	0.649	0.078–5.395	0.689		417,398.9	0	0.992

*Abbreviations*: DFS: disease free survival; CSS: cancer-specific survival; MVB: mesenteric vein blood; PB: peripheral blood HR, hazard ratio.

### Survival outcomes according to CTC positivity in follow-up CTC assessments after surgery

**Figure 4:**
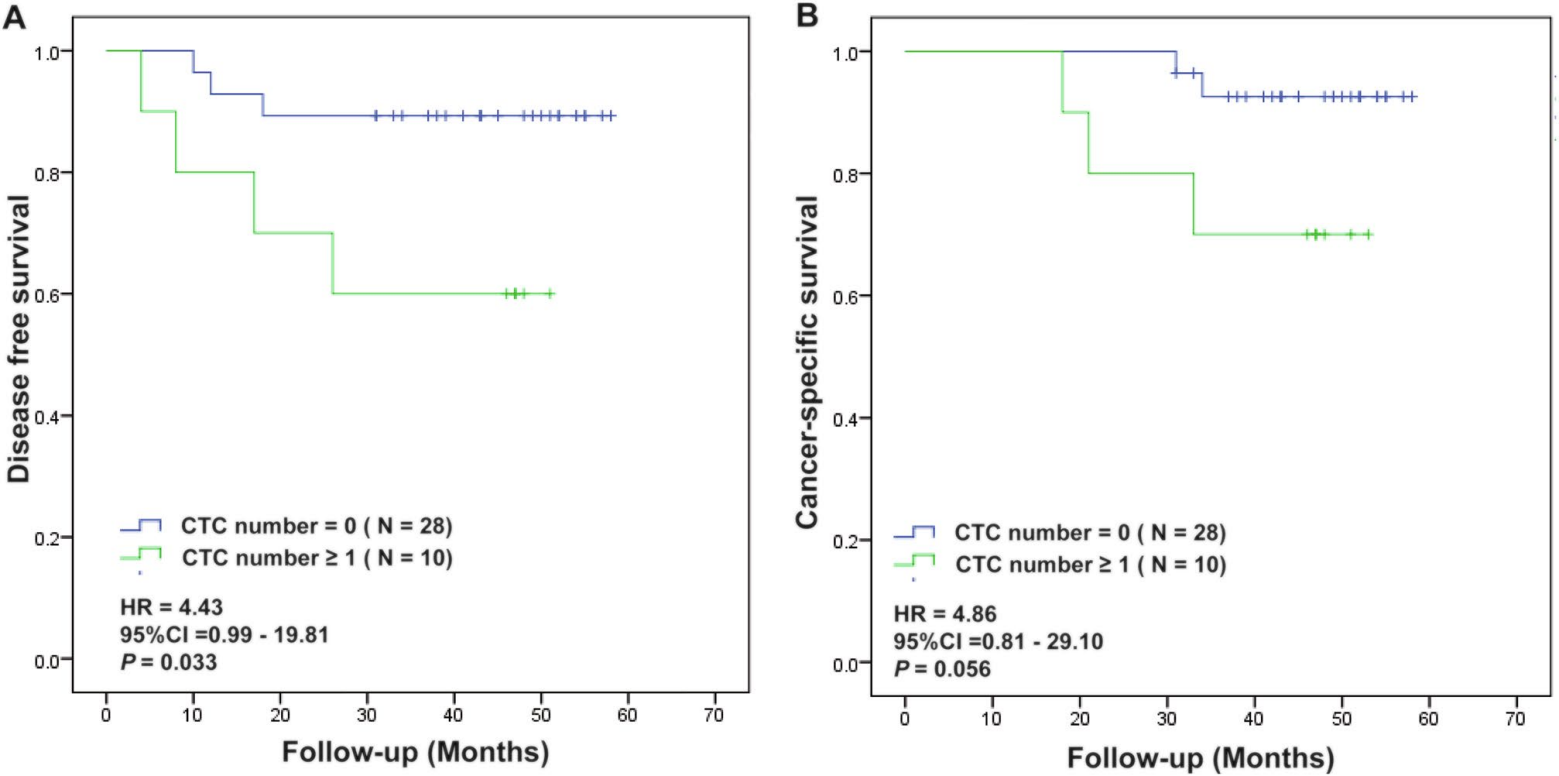
Kaplan-Meier survival analysis for DFS (**A**) or CSS (**B**) in patients with one or more CTC in PB samples after follow-up period.

To investigate the relationship between post operation CTC presence and clinical outcomes, we analyzed the survival outcomes (DFS and CSS) according to CTC positivity. In patients with follow-up CTC assessment (n = 38). The average follow-up period was 53.9 months with 7 (18.4%) cases of recurrence and 5 (13.2%) cases of death. CTC positivity demonstrated significant difference of DFS and showed a trend toward poor CSS (Fig. 4A,B). Table 3 shows the patients’ characteristics divided between patients without any CTCs and those with 1 or more CTCs in PB after surgery. There was no significant difference in the other patient characteristics according to CTC positivity.

**Table 3:** Patients' characteristics and CTCs prevalence after surgery.

Variables	Total (N = 38)	CTC present in PB (N = 10)	CTC absent in PB (N = 28)	*P* value
Median age, years (range)	65 (38–84)	57 (44–76)	66 (38–84)	
Sex, n (%)				0.4413
Male	25	8	17	
Female	13	2	11	
Location of primary tumor, n (%)				0.406
Right-side Colon	13	2	11	
Left-side Colon	12	3	9	
Rectum	13	5	8	
T stage, n (%)				0.459
T1	5	0	5	
T2	7	2	5	
T3	17	6	11	
T4	9	2	7	
N stage, n (%)				0.186
N0	20	3	17	
N1	12	4	8	
N2	6	3	3	
Stage, n (%)				0.248
I	7	1	6	
II	13	2	11	
III	18	7	11	
Vascular invasion, n (%)				0.919
Present	7	2	5	
Absent	30	8	22	
No data	1	0	1	
Lymphatic invasion, n (%)				0.709
Present	9	2	7	
Absent	28	8	20	
No data	1	0	1	
Perineural invasion, n (%)				0.096
Present	1	1	0	
Absent	36	9	27	
No data	1	0	1	
Preoperative CEA, n (%)				0.087
< 5 ng/mL	26	9	17	
≥ 5 ng/mL	12	1	11	
No data	0	0	0	
Preoperative CA-199, n (%)				0.881
< 37 IU/mL	31	8	23	
≥ 37 IU/mL	7	2	5	
No data	0	0	0	
MSI Status (%)				0.382
MSI-H	2	0	2	
MSS	32	9	23	
No data	4	1	3	
Number of nodes sampled				0.774
< 12	3	1	2	
≥ 12	35	9	26	

## Discussion

Tumor cells present in the blood reflect the invasion of the primary tumor in the bloodstream and these CTCs have the potential to give rise to distant metastasis^24^. CTCs are a potential predictor for the risk of recurrence in many types of cancers^25^. The use of CTCs was recognized as “real-time liquid biopsy” in solid tumors because it could be performed frequently, easily, and less invasively. However, CTCs are rarely found in blood, at levels typically less than 1 in a billion cells, and they are fragile. In 2004, the FDA cleared the CellSearch platform, which enriched CTCs by EpCAM targeted immunomagnetic selection, followed by manual enumeration by observation of the expression of Cytokeratin’s 4–6, 8, 10, 13, 18 and 19, lack of CD45 expression, presence of a nucleus and cell like morphology^26^. While demonstrating prognostic utility for several cancer types, CellSearch was limited by biomarker expression level, fragility of cells, complexity of user analysis, and cost^27^. CellSearch was utilized by Cohen et al. to define unfavorable prognostic groups in metastatic CRC patients by detection of ≥ 3 CTC per 7.5 mL of PB blood^9,10^. Our results show a lower detection rate of CTCs in the PB of patients with non-metastatic CRC compared to the metastatic populations previously studied. Based on the relatively low proportion of patients with ≥ 1 CTC who had a significantly worse outcome compared with patients without CTCs, the threshold of ≥ 1 CTC may be suitable for non-metastatic CRC to evaluate the risk of recurrence. The enrichment of CTCs with high purity and recovery continues to be a great challenge.

Here, we demonstrated the MiSelect R rare cell platform by evaluating its overall recovery accuracy, assay linearity, limit of blank (LOB), limit of detection (LOD), assay specificity, and precision. Our results showed that the average overall recovery rate was 91.3% ± 10.3% across the spiked cell concentration range with a 98% recovery rate when only one cell was added to 8 mL of blood. Assay specificity was determined to be 100% for 28 healthy donor blood samples. Reproducibility for intra-assay, inter-assay, and inter-operator variability had CVs of between 7 and 10% To evaluate heterogenous expression of EpCAM on CTCs, we attempted detection of EpCAM^low^ expressing MDA-MB-231 cultured cells with the MiSelect R system and we obtained a high 80% recovery rate of EpCAM^low^ CTCs. The higher efficiency for EpCAM^low^ cell recovery compared to CellSearch is likely due to the simpler and gentler sample pre-treatment combined with the higher optical sensitivity, and the lack of magnetic particles^28,29^. The validation results suggested that the MiSelect R System is suitable for rare CTC determination in non-metastatic CRC.

Primarily because of the low number of CTCs detected in early stages in PB for non-metastatic CRC patients, there is not strong reported evidence suggesting that CTC number correlates with disease recurrence. In this study, we found CTC numbers are significantly higher in mesenteric vein blood (MVB) than in peripheral blood (PB). PB is collected from a vein in the arm, and any CTCs in this vein likely traveled from a tumor through several systems which may have reduced their number, such as the liver and lungs. Finding a higher number of CTCs in MVB is consistent with a simpler path from the tumor to the MVB. While MVB collection is not possible for many patients, the collection of MVB during surgery provided a valuable sample for analysis. The preoperative presence of CTCs in MVB correlated with T staging but did not correlate with other clinicopathological factors. Results also showed a significant correlation with worse DFS and CSS when CTCs were found before surgery in the MVB. The presence of CTCs in PB during follow-up testing after surgery also showed worse prognosis than those patients without CTCs. These results demonstrated that the presence of CTCs is associated with disease recurrence.

Given the low levels of CTCs in PB of most non-metastatic CRC patients, ctDNA analysis has become more common. It is more easily detected in plasma^30^. Many studies have demonstrated ctDNA as a potential prognostic marker, that negatively correlates with colorectal cancer patient survival^31^. In serval studies of non-metastatic CRC patients, the detection rate of ctDNA is greater than 73%^32,33^. ctDNA signatures are also considered as a marker of determination for high risk group to indicate adjuvant therapy in stage II colon cancer^34,35^. Other studies suggest that in stage III colon cancer, post-chemotherapy ctDNA analysis could lead to a more informed selection of patients who could benefit from additional therapeutic approaches^36^. Even through ctDNA is potential marker for prognostic and treatment decision making, detection of CTC is still crucial because CTC and ctDNA reflect biologically different aspects of disease. CTCs can provide evaluable tumor cells for phenotyping. In early and metastatic breast cancer, PIK3CA mutation detection in CTCs and plasma-ctDNA provides complementary information^37^. CTC and ctDNA will complement biopsies as diagnostic procedures and hopefully will make cancer treatment more precise.

Collectively, these data suggest CTCs as a potentially useful prognostic and predictive biomarker in non-metastatic CRC patients that may help to further stratify patient’s risk status within different stages of disease.

## Methods

### Study design (patients and samples)

One hundred and sixteen patients with colorectal cancers and six patients with tubular adenoma before undergoing curative surgical resection were prospectively enrolled at Taipei Veterans General Hospital (VGHTPE) between October 2016 to September 2017. None of patients were diagnosed due to obstructive disease. 28 healthy controls with no current illnesses or history of any neoplastic disease were also enrolled. This study was approved by the Institutional Review Board of VGHTPE (2016-07-005CC).All patients gave written informed consent and research was conducted according to the principles expressed in the Declaration of Helsinki.

8 mL Peripheral blood (PB) and 8 mL mesenteric vein blood (MVB) samples were collected during operation. For a subset of thirty-eight patients, 8 mL PB was also collected after each follow-up visit after surgery. To determine the risk factors for CTC positivity after curative CRC surgery, we compared the preoperative patient characteristics (sex, age, and preoperative CEA level), intraoperative findings (tumor location and tumor size), and postoperative pathological characteristics (vascular invasion, lymphatic invasion, perineural invasion). The staging evaluation was performed according to the American Joint Committee on Cancer tumor-node-metastasis (TNM) staging for CRC (seventh edition). All patients were followed up for an average period of 63.2 months (range 1–65.4 months). Cancer-specific survival (CSS) was defined as the period from surgery to death from CRC-related deaths, and disease-free survival (DFS) was defined as the period from surgery to the first event of either CRC relapse or death. Patients who did not experience relapse or who died (for DFS) or remained alive (for CSS) at the final follow-up were censored at that time.

### CTC enumeration

The MiSelect R System (MiCareo, Taiwan) was used to quantify CTCs in 8 mL blood samples drawn from patients. Blood samples were collected in K_2_EDTA blood collection tubes, stored at room temperature, and processed within 24 h of collection, according to the manufacturer’s instructions. Briefly, whole blood was incubated with SelectSort EpCAM (MiCareo, Taiwan) for 20 min at room temperature. After incubation, the sample was washed with ISOTON II buffer to remove the unbound antibody and then loaded on the MiSelect R system. The MiSelect R system includes an optical detection system, a microfluidic active cell sorting scheme, and an on-chip filter for cell labeling and fluorescence imaging enumeration. Upon detection of fluorescently labeled cells in the whole blood, CTCs are diverted to a channel that leads to an on-chip filter, where they can be fixed, permeabilized, and labeled with confirmation antibodies prior to fluorescence imaging. CTCs are defined as cells with a 4’,6-diamidino-2-phenylindole (DAPI) positive nucleus, positive membrane staining for EpCAM, cytoplasmic staining for cytokeratin, and the absence of CD45 expression. The presence of one or more CTCs is defined as CTC positivity.

### Analytical validation design

CTC assay characteristics including accuracy, linearity, limit of blank (LOB), limit of detection (LOD), and precision were performed using contrived samples. To more closely mimic the cancer cells expected from patients, SkBr3, a breast cancer cell line, was chosen as a CTC surrogate for this assay validation. SkBr3 is an epithelial cancer cell line derived from human breast adenocarcinomas and has stable expression of EpCAM^38,39^. To measure assay performance characteristics, the SkBr3 cellswere spiked into healthy donor blood via exact number capillary counting. Sample processing and CTC enumeration of spiked samples followed the protocol described in the “CTC enumeration” section.

### Cell lines, cell recovery, limit of detection, precision, and linearity experiment

Sk-Br-3 and MDA-MB-231 cells were used as circulating tumor cell surrogates. Cells were cultured at 37 °C with 5% CO2 in McCoy's 5a media and DMEM media (Gibco; Thermo Fisher Scientific, Inc., Waltham, MA, cat. no 11875093 and 10566016) and supplemented with 10% fetal bovine serum and 1% penicillin/streptomycin (Gibco; Thermo Fisher Scientific, Inc., cat. no 14140-122).

Exactly number of cells were added via capillary counting to 8 mL healthy donor blood. The blood was then incubated with SelectSort EpCAM before being loaded into MiSelect R system with a SelectChip Dual (MiCareo, Taiwan) microfluidic cartridge for automated enrichment, reagent labeling, and fluorescence imaging. The cell recovery was calculated as the number of isolated cells divided by the number of cancer cells spiked into the sample.

### Statistical analysis

The association between CTC positivity and various clinicopathological characteristics was analyzed using a Fisher exact test or χ^2^ test for categorical variables and a Student t test for quantitative variables. Inferred analysis of the two population distribution patterns used the Mann–Whitney test. Kaplan–Meier survival curves for survival outcomes (DFS and CSS) according to CTC positivity were constructed with available clinical data, and the differences were evaluated using a log-rank test. Statistical significance was set at *P* < 0.05. Not all stratified groups have reached statistical significance, potentially due to the population size and completed follow up time period.

## Supplementary Information

Supplementary Information.

## Data Availability

The datasets generated during and/or analyzed during the current study are available from the corresponding author on reasonable request.
