# Supplementary material for: MiSelect R System: the validation of a new detection system of CTCs and their correlation with prognosis in non-metastatic CRC patients
Source: Sci Rep. 2023 Mar 23;13:4773. doi: 10.1038/s41598-023-31346-9 (PMC10036555; doi:10.1038/s41598-023-31346-9)
Supplement: Supplementary file 1 — Supplementary Information. [file 41598_2023_31346_MOESM1_ESM.pdf]

Validation of a Rare Cell Analysis System with Survival of Non-metastatic Colorectal Cancer Patients with Circulating Tumor Cells

Chun-Chi Lin[1], Chih-Yung Yang[2,+], Tzu-Chao Hung[3,+], Chun-Hung Wang[3], Sheng-Wen Wei[3], Perry Schiro[3], Ju-Yu Tseng [3,*], Chi-Hung Lin[4,5,6], Jeng-Kai Jiang[1,7,*]

1 Division of Colon & Rectal Surgery, Department of Surgery, Taipei Veterans General Hospital, Taipei 112, Taiwan.

2 Department of Teaching and Research, Taipei City Hospital, Taipei City,104, Taiwan

3 MiCareo Taiwan Co., Ltd., Taipei City,114, Taiwan

4 Department of Biological Science and Technology, National Yang-Ming Chiao-Tung University, Hsinchu, 300, Taiwan

5 Institute of Microbiology and Immunology, National Yang-Ming Chiao-Tung University, Taipei City, 112, Taiwan

6 Cancer Progression Research Center, National Yang Ming Chiao-Tung University, Taipei City, 112, Taiwan

7 School of Medicine, National Yang-Ming Chiao-Tung University, Taipei City, 112, Taiwan

[+] Equal contribution

*Corresponding author

Address correspondence to:

Ju-Yu Tseng, MiCareo Taiwan Co., Ltd., Taipei, Taiwan; 5F., No.69, Ln. 77, Xing Ai Rd., Neihu Dist., Taipei City, Taiwan. 114 Phone: +886-2-27923976 #301; Fax: +886-2-27965302; E-mail: jytseng@micareo.com

Jeng-Kai Jiang, Taipei Veterans General Hospital Department of Surgery, Division of Colorectal Surgery; 201, Shipai Rd. Sec. 2, Beitou Dist., Taipei City, Taiwan 112 Phone: +886-2-28757054#107; Fax: +886-2-28757639; E-mail: jkjiang@vghtpe.gov.tw

Supplemental Table 1 Analytical Results for CTC enumeration assays on MiSelect R System.

Overall recovery efficiency across the entire spiked cell range.

Number of spike-in cell	Test number	Mean number of cells recovered	Average recovery (%)	Standard deviation (%)	Coefficient of variation (%)
1	50	0.98	98%	14%	14.3%
8	6	7.5	93.8%	6.3%	6.7%
32	66	27.9	87.2%	7.6%	8.7%
128	6	112.5	87.9%	6.5%	7.4%
512	6	479.5	93.7%	4.4%	4.7%
1024	60	920	89.9%	7.0%	7.8%

LoD and LoB

Number of spike-in cell	Test number	% of positive
0	28	0%
1	44	97.7%
2	9	100%
3	21	100%

Precision

Number of cell spike-in	Test number	Average recovery (%)	Coefficient of variation (%)
1024	60	89.9%	7.8%
32	60	87.1%	9.2%

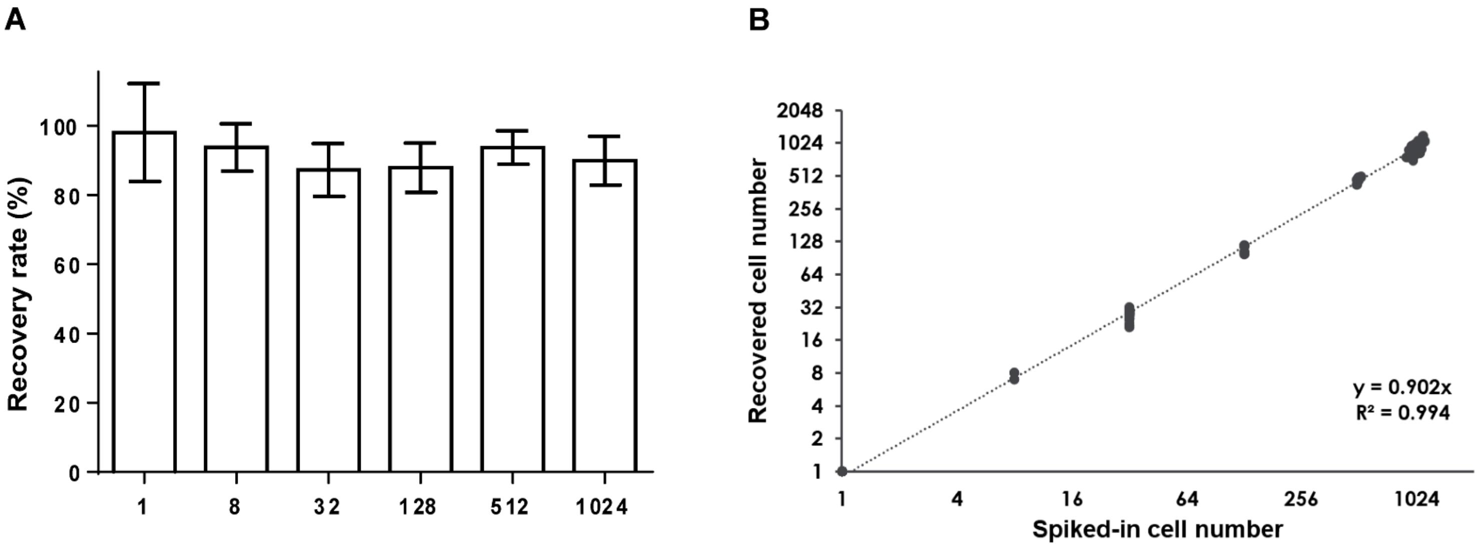

Supplemental Figure 1 SkBr3 recovery rate across the entire spiked cell range.

A B

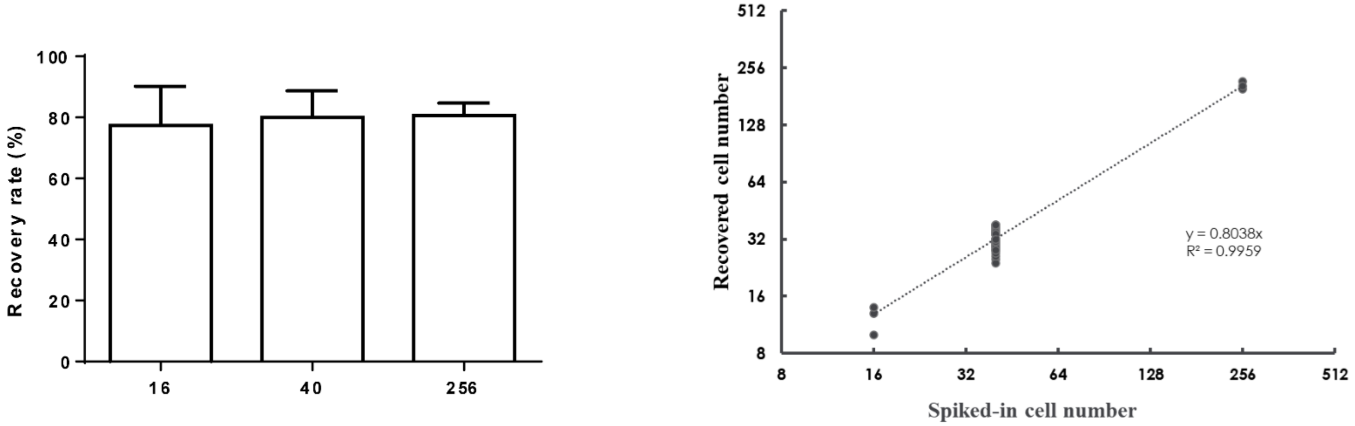

Supplemental Figure 2 MDA-MB-231 recovery rate across the entire spiked cell range.
